# Supplementary figures and images for: In silico prediction of protein-protein interactions in human macrophages
Source: BMC Res Notes. 2014 Mar 17;7:157. doi: 10.1186/1756-0500-7-157 (PMC4003812; doi:10.1186/1756-0500-7-157)

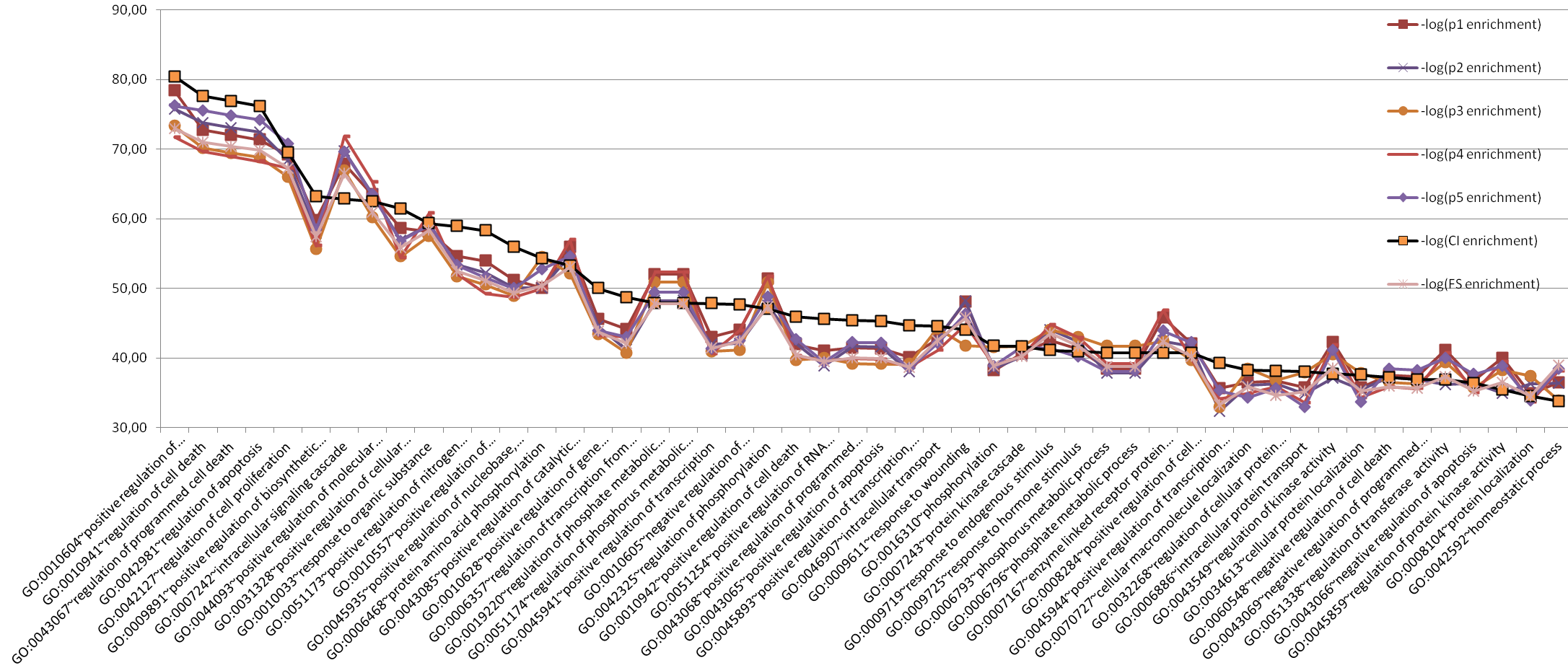

Supplement: Additional file 4: Figure S2 — Top 50 comparison enrichment terms p-values between CI and five randomised CI(s): The CI enrichments p-values (black line) are more enriched than the observed randomised CI enrichments p-values (p1, p2, p3, p4 and p5). T-test comparisons were performed between the CI and each randomised set of interactions (p1 to p5). The difference remains significant in each case with t-test p-values varying from 4.166e-05(p2) to 0.03316(p1). [file 1756-0500-7-157-S4.png]

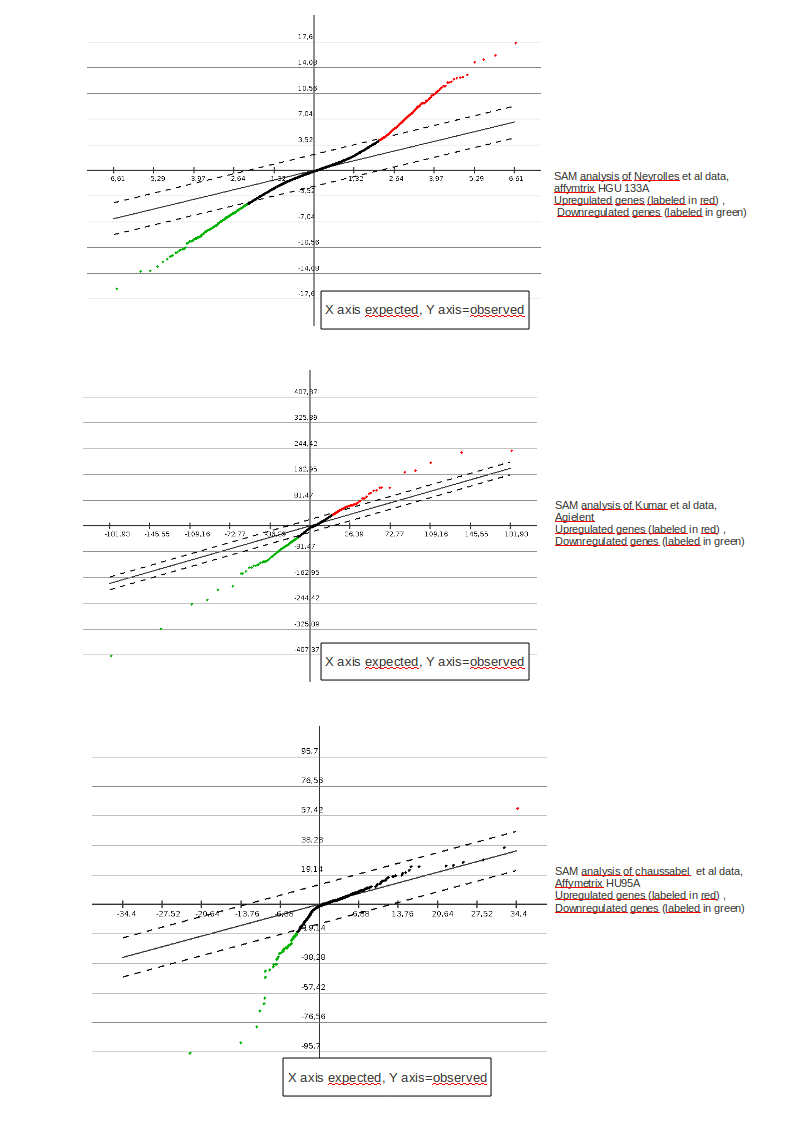

Supplement: Additional file 5: Figure S1 — Constitution of down-regulated and up-regulated gene sets. These genes were identified through SAM analysis (Significance analysis of microarray) with respect to median false discovery rate of 1%. Red points correspond to up-regulated genes and green points correspond to down-regulated genes. Top analysis [37]; Medium analysis [31]; Bottom analysis [36]. Ultimately these analyses allowed respectively the constitution of respectively 3724 and 1651 up-regulated and down-regulated gene sets. [file 1756-0500-7-157-S5.png]
